# Functional conservation and coherence of HIV-1 subtype A Vpu alleles

**DOI:** 10.1038/srep44894

**Published:** 2017-03-20

**Authors:** Bizhan Romani, Amirarsalan Kavyanifard, Elham Allahbakhshi

**Affiliations:** 1 Cellular and Molecular Research Center (CMRC), Faculty of Medicine, Ahvaz Jundishapur University of Medical Sciences (AJUMS), Ahvaz, 61357-15794, Iran; 2 Department of Biology, Faculty of Science, University of Isfahan, Isfahan, 81746-73441, Iran; 3 Department of Biology, Payam Noor University, Tehran, 1395-4697, Iran

## Abstract

Functional studies of HIV-1 proteins are normally conducted using lab adapted strains of HIV-1. The extent of those functions in clinical strains is sometimes unknown. In this study, we amplified and sequenced HIV-1 Vpu from 10 Iranian patients infected with HIV-1. Phylogenetic analysis indicated that the Vpu alleles were closely related to the CRF35_AD from Iran and subtype A Vpu. We addressed some of the well-established functions of the HIV-1 Vpu, as well as some of its recently reported functions. Ability of the clinical strains of subtype A Vpu alleles for downregulation of CD4 was similar to that of the lab adapted NL4.3 Vpu. Majority of the subtype A Vpu alleles performed stronger than NL4.3 Vpu for downregulation of SNAT1. The Vpu alleles differentially induced downregulation of HLA-C, ranging from no effect to 88% downregulation of surface HLA-C. Downregulation of tetherin and enhancement of virus release was similar for the subtype A Vpu alleles and NL4.3. Subtype A Vpu alleles were more potent when compared with NL4.3 for inhibition of NF-κB activation. Our study shows that subtype A Vpu alleles exert the classical functions of HIV-1 Vpu.

Viral protein U (Vpu) is a small accessory protein unique to HIV-1 and closely related lentiviruses^1^. Vpu was long documented to enhance release of the progeny viruses. In the absence of Vpu, viral progenies accumulate on the plasma membrane of human cell lines, such as HeLa cells^2^,^3^. However, there are cell lines in which Vpu is dispensable for virus release. This discrepancy in cell line dependency of Vpu prompted the research for finding a potential restriction factor responsible for restricting HIV-1 virion release. This restriction factor was identified as a membrane protein, tetherin, also called CD317 or BST2^4^. Tetherin is an interferon-induced restriction factor that inhibits release of many enveloped viruses by tethering the newly formed virions to the cell membrane. Vpu was shown to antagonize the antiviral role of tetherin by direct binding to tetherin and displacing it from the site of viral assembly. Vpu-induced degradation of tetherin also reportedly plays a role in antagonizing tetherin^5^. Downregulation of tetherin by Vpu also inhibits secondary anti-HIV responses, such as interferon production and antibody-dependent cellular cytotoxicity that are mediated by tetherin^6^,^7^. Suppression of anti-HIV responses by Vpu, however, should not be solely attributed to tetherin antagonism. Vpu has been shown to inhibit activation of NF-κB independently of its anti-tetherin activity by preventing nuclear translocation of the p65 subunit of NF-κB^8^.

In addition to tetherin, Vpu also downregulates CD4 molecules, which serve as the specific viral receptors. Intracellular CD4 molecules interact with HIV-1 glycoprotein gp160 in the endoplasmic reticulum and block gp160 cleavage and maturation. It is believed that Vpu-mediated degradation of CD4 contributes to the release of envelope precursors into the normal maturation pathway^9^,^10^,^11^. A recent study demonstrated that *de novo* expression of Vpu resulted in downregulation of the neutral amino acid transporter SNAT1 on plasma membrane of activated primary human CD4+ T cells. It was found that in order to induce degradation of SNAT1, Vpu takes advantage of the same cellular machinery used for antagonizing tetherin. It was hypothesized that antagonism of SNAT1 by HIV-1 Vpu interferes with amino acid metabolism which is required for primary CD4+ T cell mitogenesis^12^.

Different variants of HIV-1 proteins from different subtypes demonstrate different activity levels. In addition, various proteins of the same subtypes carrying naturally occurring mutations could also act differently^8^,^13^,^14^. Majority of the functional studies of HIV-1 proteins have been conducted on lab adapted clones of HIV-1, such as NL4.3, that do not represent characteristics of clinical strains or other HIV-1 subtypes. In this study, we addressed HIV-1 Vpu alleles isolated and cloned from clinical strains of Iran. Phylogenetic analysis of the Vpu alleles showed that they cluster with HIV-1 subtype A and the previously reported CRF35_AD from Iran. Functional analysis of the Vpu alleles showed that they form a coherent group with similar activities despite sequence differences.

## Results

### Phylogenetic analysis of the clinical Vpu alleles

**Figure 1:**
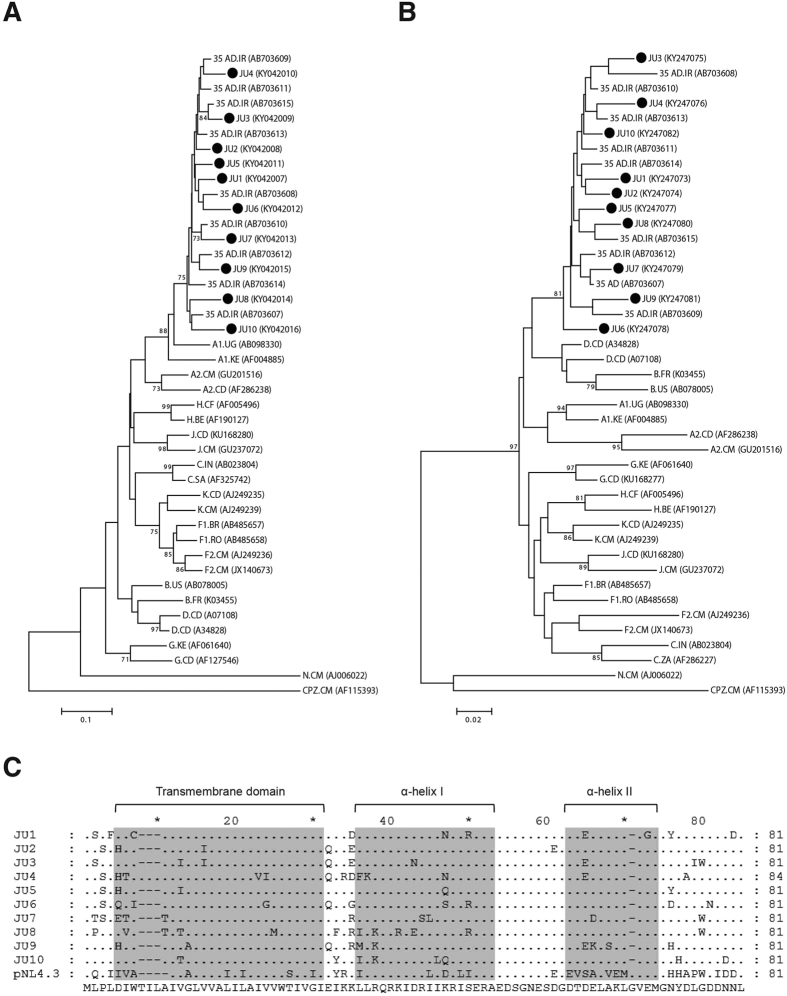
Phylogenetic analysis of HIV-1 isolates. (**A**) DNA sequences of the Vpu alleles were aligned with Vpu of HIV-1 group M reference sequences. A phylogenetic tree was constructed and rooted using Vpu sequences of HIV-1 group N and SIVcpz. Ten Vpu alleles of the clinical isolates are shown with JU (Jundishapur University) suffixed by their respective clone number (1 to 10). For clarity, our isolates are indicated with filled circles (•). Gene accession numbers are indicated in brackets and the branch lengths represent the sequence dissimilarities. (**B**) A phylogenetic tree was constructed with partial Pol DNA sequences of the HIV-1 isolates and the reference sequences of HIV-1 group M. The tree was rooted with HIV-1 group N and SIVcpz. (**C**) Alignment of the deduced Vpu amino acid sequences from 10 Iranian isolates along with the NL4.3 Vpu. Dots represent identity to the sequence at the bottom and dashes represent gaps. Transmembrane and α-helical domains of Vpu are shaded gray.

Previous studies of the HIV-1 strains isolated from Iran have reported prevalence of a circulating recombinant form (CRF) of subtype A and D, known as CRF35_AD. The majority of CRF35_AD sequence shows homology with subtype A, but it also has traces of non-subtype A sequences^15^,^16^. In order to characterize and clone HIV-1 Vpu, we amplified and sequenced Vpu fragments from PBMCs of 10 HIV-1 infected individuals from Iran. The patients’ characteristics and the gene accession numbers are listed in Table 1. Phylogenetic analysis of the sequences indicated that all the Vpu alleles clustered with the previously described isolates of CRF35_AD (Fig. 1A). Furthermore, this cluster was closely related to HIV-1 subtype A from other regions of the world.

In order to certainly determine subtypes of new isolates, sequences of other regions of the viral genomes are required. Hence, we sequenced a fragment of HIV-1 Pol that reportedly displays a subtype D recombinant origin in CRF35_AD^17^. Phylogenetic analysis of partial Pol sequences indicated that our isolates were closely related to the previously described CRF35_AD isolates of Iran (Fig. 1B). Interestingly, the cluster containing our isolates and the previously reported CRF35_AD isolates were related to subtype B and D. Subtype B and D share the highest homology among all HIV-1 subtypes and phylogenetic studies have found that subtype B sequences cluster as part of a larger subtype D cluster^18^,^19^,^20^. Taken together, our isolates share fragments of different subtypes and therefore they represent recombinant forms of HIV-1 subtypes related to CRF35_AD isolates from Iran.

The *vpu* gene is located in a region of CRF35_AD that was originally obtained by recombination of a parental sequence of subtype A. Hence, our Vpu alleles, which also showed a high homology with CRF35_AD and subtype A, represent subtype A Vpu. Alignment of the amino acid sequences of the Vpu alleles indicated that all the isolated Vpu alleles contained amino acid substitutions in different regions (Fig. 1C). Except for one isolate, JU4 with 84 amino acids in length, all the other 9 isolates displayed 81 amino acids in length. Many of the amino acid substitutions occurred in more than one allele, suggesting the prevalence of those substitutions among clinical isolates. A SIFT analysis of the amino acid substitutions suggested that the amino acid variations were tolerated by the subtype A Vpu alleles (SIFT score >0.05) and no deleterious mutation was detected among the alleles (Supplementary Table S1).

### Downregulation of protein targets by subtype A Vpu

**Figure 2:**
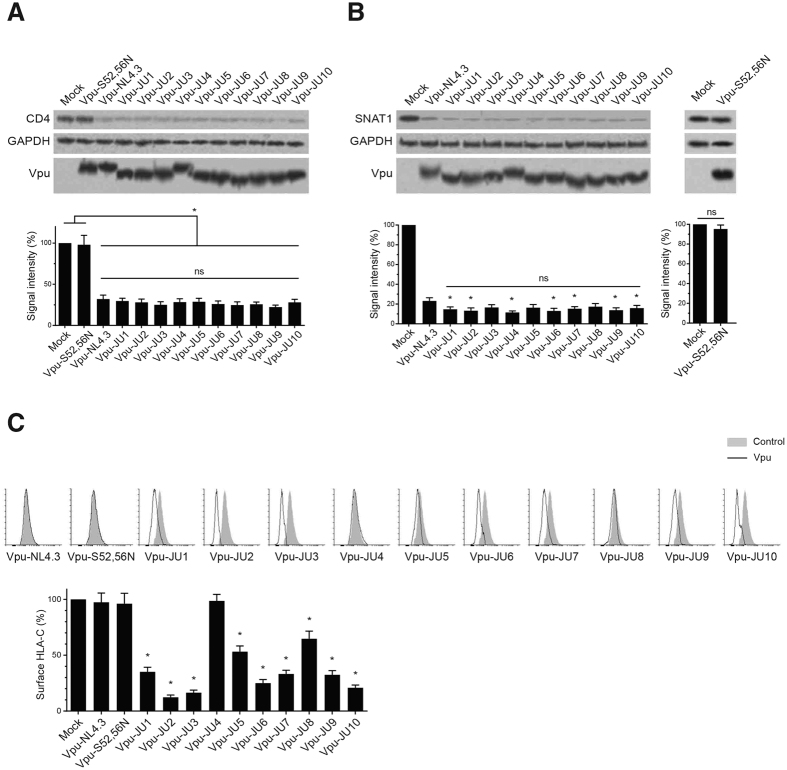
Downregulation of cellular proteins by HIV-1 Vpu alleles. (**A**) CEM-CD4^+^ cells were transduced with GFP-marked lentiviral vectors expressing Vpu alleles. A mutant form of Vpu (Vpu-S52, 56 N), incapable of downregulating CD4, was used as control. After 48 h, transduced cells were sorted using the GFP signal and subjected to Western blot to assess CD4 expression level by Vpu alleles (n = 3). (**B**) CEM-CD4^+^ cells were transduced with GFP-marked lentiviral vectors expressing Vpu alleles. GFP-positive cells were sorted after 48 h and analyzed using Western blot to assess downregulation of SNAT1 by Vpu alleles (n = 3). Asterisks indicate significant differences of subtype A Vpu alleles compared with NL4.3 Vpu. (**C**) Vpu alleles were tested for their ability to downregulates HLA-C on the cell surface of CEM-CD4^+^ cells transduced with GFP-marked lentiviral vectors. The bar graph shows summary of 3 independent experiments. Asterisks indicate significant differences of subtype A Vpu compared with NL4.3 Vpu.

Expression of Vpu is well documented to downregulate a number of proteins. In order to test the functionality of Vpu alleles, we tested downregulation of a number of Vpu protein targets. CD4 is a well-established protein target whose degradation is induced by Vpu. This function of Vpu has been extensively explored in the context of subtype B Vpu. However, the occurrence and the extent of Vpu-induced CD4 downregulation in other subtypes is not sufficiently addressed. In this study, we expressed subtype A Vpu alleles in CEM-CD4^+^ cells and assessed downregulation of the endogenous CD4 molecules (Fig. 2A). All of the subtype A Vpu alleles effectively downregulated CD4 molecules, ranging from 24.7 to 29.6%. Similarly, Vpu-NL4.3, reduced CD4 expression to 32.1% which was comparable to the ability of subtype A Vpu alleles for downregulation of CD4 molecules. The mutant S52, 56 N, which is impaired for β-TrCP binding, was unable to downregulate CD4. No significant difference was found among the clinical isolates compared with one another or the wild-type Vpu-NL4.3.

Vpu has recently been reported to induce degradation of SNAT1. In order to examine this function among the subtype A Vpu alleles, CEM-CD4^+^ cells were transduced with lentiviral vectors expressing Vpu alleles (Fig. 2B). Expression of all subtype A Vpu alleles resulted in SNAT1 downregulation, ranging from 13.0 to 17.4%. This suggested conservation of SNAT1 downregulation among the clinical strains of subtype A Vpu alleles. Interestingly, expression of Vpu-NL4.3 reduced SNAT1 expression level to 23.2%. Downregulation of SNAT1 by the majority of the subtype A Vpu alleles was more efficient than that by Vpu-NL4.3. Interestingly, expression of the mutant form of Vpu (S52, 56 N) did not induce downregulation of SNAT1.

A recent study reported that clinical strains of HIV-1 Vpu mediate downregulation of HLA-C possibly to evade CTL recognition^21^. We tested the effect of Vpu alleles on the surface expression of HLA-C and found that subtype A Vpu alleles show a wide range of effects against HLA-C (Fig. 2C). Vpu-NL4.3, Vpu-S52, 56 N, and Vpu-JU4 did not affect the surface expression of HLA-C while all other alleles showed some level of activity against HLA-C. A consensus sequence of the examined subtype A Vpu alleles is shown in Supplementary Fig. S1. The mutations associated with inability to downregulate HLA-C are indicated. Vpu-JU4 and Vpu-NL4.3, both of which were defective in HLA-C downregulation, shared two mutations at position 31 and 74. Those mutations were not found in other alleles.

The highest level of HLA-C downregulation was induced by Vpu-JU2 that reduced the surface expression of HLA-C to 12.3%. This Vpu allele had a relatively low number of amino acid substitutions throughout its sequence compared with the consensus sequence of the Vpu alleles (Fig. 1C). Especially, the α-helix-I and II of JU2 were free of such amino acid variations that could affect its activity.

A summary of all the measured values is shown in Supplementary Table S2. Taken together, the majority of subtype A Vpu alleles efficiently downregulated HLA-C. However, a comparison between subtype A and B was not possible since NL4.3, as previously also reported, does not represent the typical activity of the clinical strains of subtype B for HLA-C downregulation^21^.

### Downregulation of tetherin and facilitating virus release by subtype A Vpu

**Figure 3:**
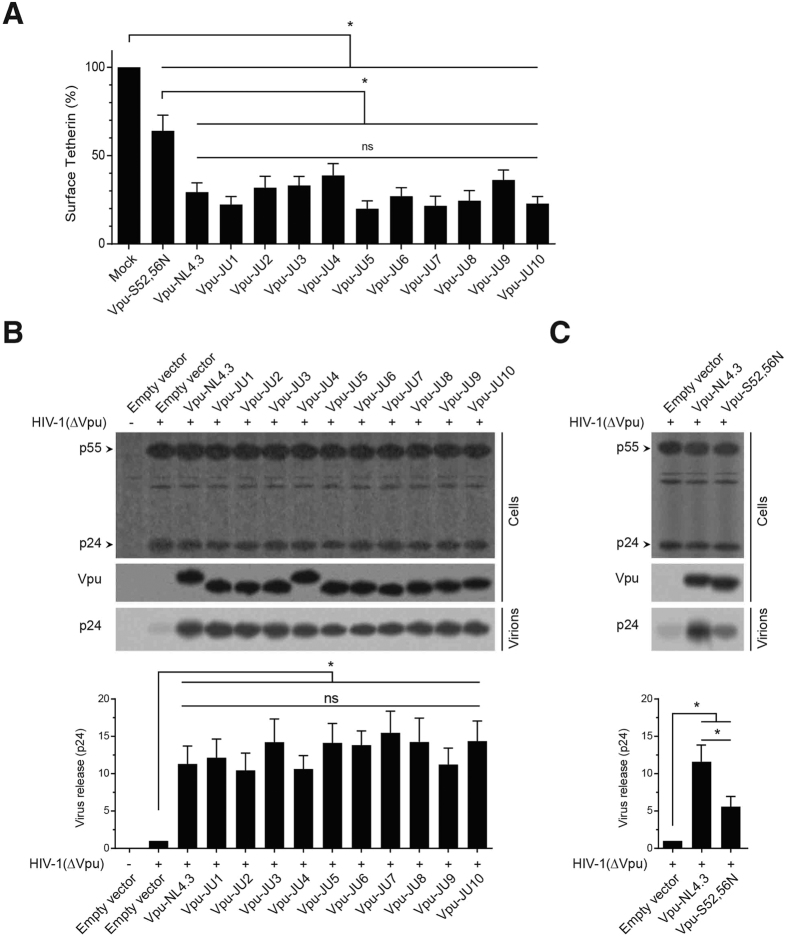
Downregulation of surface tetherin and enhancing virus release by Vpu alleles. (**A**) CEM-CD4^+^ cells were transduced with GFP-marked lentiviral vectors for expression of Vpu alleles. After 48 h, surface expression of tetherin was measured in the GFP-positive transduced cells (n = 3). (**B**) HeLa cells were cotransfected with HIV-1 ∆Vpu/Nef and expression vectors for expression of Vpu alleles. After 48 h, cell lysates and supernatants were analyzed using Western blot. Fold increase in virus release was calculated by quantifying the signal density of cell-free p24 in the supernatant. (**C**) HeLa cells were cotransfected with HIV-1 ∆Vpu/Nef and expression vectors for expression of the WT Vpu (NL4.3) or the mutant Vpu (S52, 56 N). Cell lysates and supernatants were analyzed as described for B.

Tetherin is a well-established protein target of Vpu whose surface downregulation by Vpu enhances virus release from the infected cells. In this study, we found that expression of subtype A Vpu alleles in CEM-CD4^+^ cells significantly reduced the amount of membrane-associated tetherin (Fig. 3A). Although the effects of subtype A Vpu alleles were variable among the isolates, ranging from 19.9 to 38.7%, they were still comparable to that of Vpu-NL4.3, 29.3%.

We did not find a significant difference between the abilities of subtype A Vpu alleles and NL4.3 for tetherin downregulation. Vpu-induced downregulation of surface tetherin correlates with virus release from the infected cells. In order to assess the effect of Vpu on virus release, Vpu alleles were cotransfected with an HIV-1 ∆Vpu/Nef construct in a complementation experiment (Fig. 3B). Despite their differences in downregulation of surface tetherin, all Vpu alleles comparably enhanced the virus release to the same levels, ranging from 10.4 to 15.5 fold increase (also see Supplementary Table S2). Complementation with Vpu-NL4.3 resulted in 11.3 fold increase in the level of cell-free p24, suggesting that there was no significant difference between subtype A and B Vpu in terms of their abilities to enhance virus release. The Vpu mutant S52, 56 N, which is defective for CD4 downregulation, showed reduced ability for virus release (Fig. 3C). Although the extent of virus release by this mutant was still significantly higher than the mock-transfected sample, the mutant form of Vpu did not enhance the virus release to the level of the WT Vpu.

### Inhibition of NF-κB activation by subtype A Vpu

**Figure 4:**
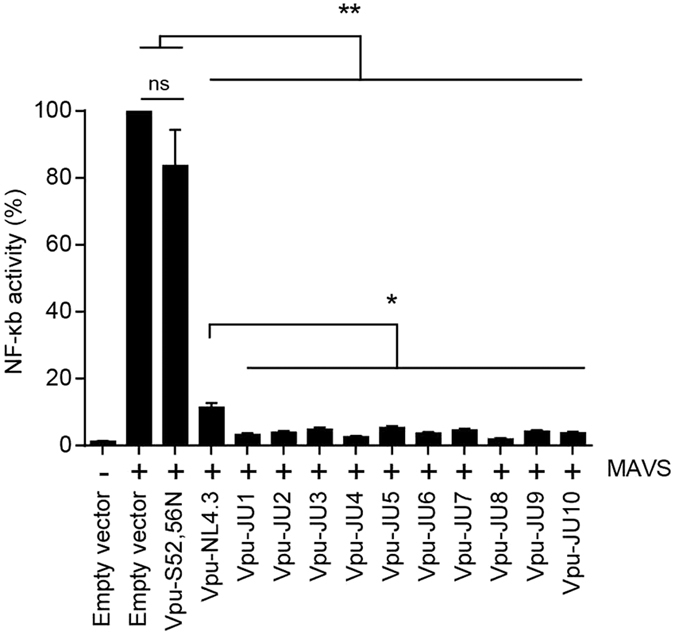
Inhibition of NF-κB by Vpu alleles. HEK293T cells were cotransfected with a vector for expression of MAVS as inducer of NF-κB and a firefly luciferase reporter construct controlled by five NF-κB binding sites. After 12 h, cells were transduced with lentiviral vectors for expression of Vpu alleles or empty vector as control (n = 3). Luciferase activities were measured 36 h post-transduction. Luciferase activity of the empty vector in the presence of the MAVS inducer was set on 100% as the control. Luciferase activities of other samples were calculated based on the control.

NF-κB plays a key role in expression of proinflammatory genes such as cytokines, chemokines, and adhesion molecules^22^. Viruses generally tend to curb those responses and HIV-1 is no exception. Among different mechanisms adopted by HIV-1, Vpu is well described to inhibit NF-κB activation. To explore this, we investigated NF-κB activation by mitochondrial antiviral-signaling protein (MAVS), an intermediate protein in the RIG-I dependent activation pathway of NF-κB. Vpu alleles were transduced into HEK293T cells expressing a luciferase expression vector under the control of 5 copies of NF-κB binding site and MAVS expression vector (Fig. 4). In the absence of Vpu, NF-κB was effectively activated and resulted in expression of luciferase from the NF-κB controlled vector. However, when subtype A Vpu alleles were expressed in HEK293T cells, they effectively inhibited NF-κB activation such that only 1.9 to 5.3% of the NF-κB activity was detectable. Although Vpu-NL4.3 also reduced the NF-κB activity to 11.4%, ability of the subtype A Vpu alleles was significantly greater than that of Vpu-NL4.3 (Fig. 4, also see Supplementary Table S2). These results suggested a functional difference between subtype A and NL4.3 Vpu in their abilities for suppression of the immune response.

## Discussion

HIV-1 reverse transcriptase is an error-prone enzyme that causes a high mutation rate in the virus genome^23^. Such mutations could translate into amino acid sequences of HIV-1 proteins and have functional consequences. Different regions and proteins of HIV-1 have different mutation rates^24^. Variability of the protein sequences among different subtypes and among different alleles of the same subtype cause functional differences between proteins^13^,^14^. A comparison of Tat proteins of nine HIV-1 subtypes showed that subtype C Tat was defective for monocyte chemotactic activity while Tat proteins of other subtypes stimulated monocyte migration^25^. The study of Vif proteins from different subtypes showed that depending on the subtype, Vif proteins differ in their abilities to counteract restriction of different APOBEC3 proteins^26^. A comparison of Nef alleles of clinical strains of subtype A, B, C, and D showed that subtype B Nef proteins displayed a greater CD4 and HLA downregulation activity while subtype C Nef proteins displayed the lowest *in vitro* activity^27^. All these studies point at functional differences between the proteins of different HIV-1 subtypes and stress the importance of addressing those differences.

We studied the clinical subtype A Vpu alleles to find out whether subtype A Vpu proteins exert the previously reported functions of Vpu. More specifically, because most functional analyses have been conducted using HIV-1 subtype B clones, we compared our results with NL4.3 (subtype B) Vpu. We found that subtype A Vpu alleles displayed similar levels of CD4 and SNAT1 downregulation. Ability of subtype A and NL4.3 Vpu for downregulation of CD4 was comparable but subtype A displayed a greater SNAT1 downregulation activity. SNAT1 downregulation by Vpu has recently been reported and its extent among clinical strains has not been addressed yet.

HIV-1 Nef is well-known for its ability to specifically downregulate HLA-A and -B^28^,^29^. But the ability of HIV-1 to downregulate HLA-C was unknown. Only recently was it demonstrated that HIV-1 Vpu induces HLA-C downregulation^21^. We tested subtype A Vpu for its ability to downregulate HLA-C. We found a broad range of HLA-C downregulation that ranged from no activity by Vpu-JU4 to a significant reduction in the levels of membrane HLA-C. Similarly to the previous report, NL4.3 Vpu did not affect levels of HLA-C. Vpu-JU4 and Vpu-NL4.3, both shared 2 similar mutations that were not found in other alleles. An arginine at position 34 and an alanine at position 77 of JU4 replaced lysine and aspartic acid, respectively. The same mutations were found in NL4.3. The defect in both proteins may be resulted from the mutation that they shared or different mutations. For instance, JU4 had 3 additional amino acids in its transmembrane domain that may affect its activities. The detailed mechanism of HLA-C Vpu downregulation by Vpu is not described yet and the involved domains of the protein are unknown. Despite the uncharacterized mechanism, our results confirm the recent findings and demonstrate the widespread ability of subtype A Vpu for HLA-C downregulation.

A recent study compared subtype B and C Vpu proteins for downregulation of CD1d, a membrane protein downregulated by Vpu, and found that subtype C Vpu proteins were poor CD1d antagonists compared with subtype B Vpu proteins. The study identified a C-terminal APW motif in subtype B alleles that was essential for downregulation of surface CD1d^30^.

Several studies that compared Vpu proteins of different subtypes reported that subtype C Vpu exhibits reduced enhancement of viral release compared with subtype B. This reduction also correlated with the reduced anti-tetherin activity of subtype C Vpu^31^,^32^. The literature lacks addressing the role of subtype A Vpu in downregulation of tetherin and enhancement of virus release. Here we studied this function of subtype A Vpu and found that the subtype A Vpu alleles display comparable potential for downregulation of surface tetherin. Furthermore, subtype A Vpu functioned to a level comparable to that of NL4.3 Vpu. Similarly, comparison of subtype A alleles with NL4.3 Vpu indicated that they enhance viral release to similar levels.

A recent study addressed the inhibitory effect of Vpu on NF-κB activation using Vpu alleles of different subtypes. They showed that Vpu proteins of all subtypes counteracted NF-κB activation, however, this study did not conduct a comparative analysis of Vpu alleles since it was not within the scope of the study^8^. In our study, we found the ability of subtype A Vpu alleles for inhibition of NF-κB activation. This activity by subtype A Vpu alleles was found more potent than that of NL4.3 Vpu.

Except for HLA-C downregulation, subtype A Vpu alleles functioned similarly, despite the naturally occurring mutations in their sequences. However, the level of activities by subtype A Vpu alleles was normally distinct from NL4.3 Vpu. It should be noted that NL4.3 Vpu displays reduced activities compared with typical subtype B Vpu alleles in tetherin downregulation^13^, as well as HLA-C downregulation^21^. Although our subtype A Vpu alleles generally performed stronger than NL4.3 Vpu for the majority of functions, we cannot conclude that subtype A Vpu is more potent than subtype B alleles for the conventional functions of Vpu.

Variations in the protein activity levels of HIV-1 subtypes, could translate into differences in the course of pathogenesis, immune response, and geographical distribution. Functional analysis of HIV-1 proteins from different subtypes will help us better understand the physiological reason behind the pathology of different subtypes.

## Materials and Methods

### Ethics statement

This study was approved by the ethics committee of Ahvaz Jundishapur University of medical sciences. All HIV-1 positive individuals gave informed consent. All experiments were performed in accordance with the approved guidelines and regulations, and the experimental protocols were approved by the institutional review boards of Ahvaz Jundishapur University of medical sciences.

### Cell lines

CEM-CD4+ cells were obtained from the NIH AIDS Research and Reference Reagent Program and maintained in RPMI supplemented with 100 units/ml penicillin, 100 μg/ml streptomycin, and 10% FBS. HeLa and HEK293T cells were described before^33^,^34^.

### Antibodies and reagents

CD4 (ab133616) and p24 (ab9071) antibodies were purchased from Abcam. APC anti-human tetherin (348410) and APC mouse IgG1 isotype control were from Biolegend. HLA-C antibody (MABF233) was from Millipore. SNAT1 antibody (sc-67080) was from Santa Cruz. GAPDH antibody (14C10) was from cell signaling. Mouse and rabbit HRP-conjugated antibodies were from Abcam. Alexa Fluor 488 conjugated anti-mouse IgG2b antibody (A-21141) and mouse IgG2b isotype control (MA5-14447) were from ThermoFisher Scientific. Polyclonal antibody against a conserved region of HIV-1 Vpu (both subtype A and B) was raised in mouse by conjugating KLH (keyhole limpet hemocyanin) to a synthetic peptide (ERAEDSGNESDG) corresponding to amino acids 48–59 of Vpu.

### Sequencing and phylogenetic analysis

Blood samples were collected from 10 HIV-1 positive individuals described in Table 1. Viral RNA was extracted from the plasma samples using the QIAamp UltraSens Virus Kit (Qiagen) according to the manufacturer’s instructions and stored at −80 °C. Viral RNA was reverse transcribed into cDNA using HIV-1-env-R (5′-CTT CCT GCT GCT CCT AAG AAC CCA -3′) with SuperScript II Reverse Transcriptase (ThermoFisher Scientific). To amplify the *vpu* gene, cDNA was then amplified using HIV-1-tat-F (5′-ATT TCC TAT GGC AGG AAG AAG CGG A-3′) and HIV-1-env-R with the Promega GoTaq Flexi Kit (Promega). The primers amplified a fragment which was about 1800 nucleotides in length. To amplify the pol gene, cDNA was amplified using HIV-1-pol-F (AGG ACA CCA AAT GAA AGA CTG CAC T) and HIV-1-pol-R (CAT ATT TCT TCC AAT TAT GTT GAC A). The primers amplified a fragment which was about 460 nucleotides in length. This fragment was chosen such that it contained the previously described recombination site of CRF35_AD^17^. PCR products were sequenced using the BigDye Terminator v3.1 Cycle Sequencing Kit (ThermoFisher Scientific) and analyzed on an ABI Prism 3130 automated DNA sequencer (Applied Biosystems). Vpu (246 nucleotides) and the recombinant fragment of Pol (268 nucleotides) were trimmed and the nucleotide sequences were submitted to GenBank under accession numbers indicated in Table 1. Sequences were aligned with HIV-1 reference sequences obtained from LANL HIV Database, using Clustal X2^35^. The alignments were manually verified using BioEdit version 7.2.5^36^. MEGA version 7^37^ was used to construct a neighbor-joining phylogenetic tree with a uniform rate of variation among sites and nucleotide substitution using the Kimura two-parameter^38^. The reliability of the branching and clustering pattern was estimated from 1000 bootstrap replicates. The tree was rooted with the reference sequences of HIV-1 group N and SIVcpz.

### SIFT analysis

Effects of the single nucleotide polymorphisms (SNPs) on the protein function were analyzed using SIFT algorithm (http://sift.jcvi.org). The effect of SNPs were considered tolerated if tolerance index >0.05.

### Consensus sequences

To show the characteristics of the clinical isolates, consensus sequence of the subtype A Vpu alleles was obtained using WebLogo (http://weblogo.threeplusone.com/).

### Vectors and virus constructs

To construct the GFP-marked lentiviral vector for expression of Vpu alleles, Vpu was amplified and sequenced 3 times from separately amplified PCR products using Vpu-F (5′-AAG TTT AAA CAA TTA GTA TGT GTA ATG-3′) and Vpu-R (5′-TT G TTT AAA CTT GTC TAC AGC ACT ACA-3′). One out of the three Vpu sequences, which represented the predominant amino acid sequence of Vpu, was selected for further analysis and cloning. HIV-1 pNL4.3 was obtained from the National Institutes of Health AIDS Reagent Program and its Vpu gene was amplified as described for the Vpu alleles. The PCR products were then digested using *pme*I restriction site and ligated into the pWPI lentiviral vector. The mutant S52, 56 N was generated by replacing two serine residues (S52 and S56) at phosphorylation sites of NL4.3 Vpu with asparagine using QuikChange II Site-Directed Mutagenesis Kit as manufacturer’s instructions. psPax2, pCMV-VSV-G and pMSCV-MAVS were obtained from Addgene. HIV-1 ∆Vpu/Nef was obtained from the National Institutes of Health AIDS Reagent Program. pGL4.32[*luc2P*/NF-κB-RE/Hygro] was from Promega.

### Lentiviral vector production

VSV-G pseudotyped GFP marked lentiviral vectors were produced by transfection of HEK293T cells with 40 μg of pWPI-Vpu alleles, 15 μg psPax2 and 6 μg of pCMV-VSV-G using the standard calcium phosphate protocol in 15 cm Petri dishes. To collect the lentiviral vector particles, supernatant of the transfected cells was collected 48 and 72 h post-transfection by ultracentrifugation at 35,000 rpm for 2 h.

### Lentiviral transduction

CEM-CD4^+^ cells were transduced with the VSV-G pseudotyped lentiviral vectors using spinoculation method. Briefly, 10^6^ cells were transduced with the lentiviral vectors at an MOI of 2.0 in 24-well plates. To enhance transduction efficiency, 8 μg/ml polybrene was added to each well. Plates were centrifuged at 1500 g for 1 h at 25 °C. Cells were then transferred to 6-well plates and incubated at 37˚C for 48 h. HEK293T cells were transduced with VSV-G pseudotyped lentiviral vectors at an MOI of 2.0 by adding 8 μg/ml polybrene.

### Luciferase assay

HEK293T cells were cotransfected with pMSCV-MAVS and pGL4.32[*luc2P*/NF-κB-RE/Hygro] using the standard calcium phosphate protocol. After 12 h, cells were transduced with VSV-G pseudotyped lentiviral vectors expressing Vpu alleles. Thirty six hours after transduction, cells were washed with PBS and lysed in the tissue culture plates using 1% Triton X-100. Luciferase was measured in the cell lysates using Bright-Glo Luciferase Assay System (Promega).

### Flow cytometry and sorting

CEM-CD4^+^ cells were transduced with the VSV-G pseudotyped GFP-marked lentiviral vectors expressing Vpu alleles. After 48 h, cells were labeled with specific antibodies against HLA-C or tetherin. GFP-expressing cells were gated and analyzed for the surface expression of the proteins of interest using CyAn ADP Analyzer. In order to analyze downregulation of CD4 and SNAT1, CEM-CD4^+^ cells were transduced with VSV-G pseudotyped GFP-marked lentiviral vectors expressing Vpu alleles. After 48 h, GFP-positive cells were sorted using an Influx cell sorter (BD Biosciences) and analyzed by Western blot.

### Western blotting

Supernatant of transfected HeLa cells or 3 × 10^5^ sorted GFP-positive CEM-CD4^+^ cells were resuspended in Laemmli buffer. Cell lysates were heat-denatured for 5 min and loaded in 12% SDS-PAGE gels. Western blots were performed as described previously^33^.

### Statistical analysis

Unpaired two-tailed T test was used for the statistical analysis using GraphPad Prism 6.0. A value of *p* < 0.05 was considered statistically significant and indicated with an asterisk (*). Results were expressed as mean S.D. or S.E.M and represent data from a minimum of three independent experiments.

## Additional Information

**How to cite this article**: Romani, B. *et al*. Functional conservation and coherence of HIV-1 subtype A Vpu alleles. *Sci. Rep.* **7**, 44894; doi: 10.1038/srep44894 (2017).

**Publisher's note:** Springer Nature remains neutral with regard to jurisdictional claims in published maps and institutional affiliations.

## Supplementary Material

Supplementary File

## Figures and Tables

**Table 1:** Patients’ characteristics.

Patient	Age	Gender	Viral load^1^	CD4 counts/mm3	HAART	Transmission	Gene accession number (Vpu)	Gene accession number (Pol)
JU1	42	Male	<100	1188	Yes	Injected drug use	KY042007	KY247073
JU2	23	Male	55, 680	240	No	Heterosexual	KY042008	KY247074
JU3	51	Male	<100	1247	Yes	Injected drug use	KY042009	KY247075
JU4	26	Female	1043	879	Yes	Heterosexual	KY042010	KY247076
JU5	38	Male	<100	741	Yes	Injected drug use	KY042011	KY247077
JU6	25	Male	8740	452	No	Injected drug use	KY042012	KY247078
JU7	43	Female	371	648	Yes	Heterosexual	KY042013	KY247079
JU8	30	Female	<100	978	Yes	Heterosexual	KY042014	KY247080
JU9	29	Male	<100	1507	Yes	Heterosexual	KY042015	KY247081
JU10	22	Male	12460	547	No	Heterosexual	KY042016	KY247082

^1^Viral RNA copies per ml peripheral blood.
