# Erratum: Functional conservation and coherence of HIV-1 subtype A Vpu alleles

**DOI:** 10.1038/srep46882

**Published:** 2017-08-29

**Authors:** Bizhan Romani, Amirarsalan Kavyanifard, Elham Allahbakhshi

Scientific Reports 7: Article number: 44894; 10.1038/srep44894 published online: 03 20 2017; updated: 08 29 2017.

This article was published twice in error during a change in production systems. The publisher apologizes to the authors and readers for the error. When citing this work, please refer to the original version.^1^
